# Dysentery

**Published:** 1868-10-15

**Authors:** A. A. Dunn

**Affiliations:** Chicago


### DYSENTERY.

BY A. A. DUNN, M.D., CHICAGO.

Having, in my practice, so frequently seen inflammation of various tissues of the body subside and disappear under the sole, or very nearly the sole, administration of anodynes, my mind tends very strongly to the opinion that we may, in a vast number of cases, bedevil our patients with drugs better dispensed with than dispensed. Assuming, as we are warranted in doing, that in most forms of disease the tendency of the organism is to recovery, to health, through the eliminative and recuperative processes of the vital forces, it may be safe also to assume that recoveries under the simple use of anodynes would have ensued, in the majority of cases at least, had nothing been given. Argument, perhaps, independent of experience or observation, can hardly demonstrate that proposition; but, based upon what we daily observe, we are confirmed in its probable truth. This admission, however, does not relieve us of the impression that, in the very cases which would most probably recover if uninterfered with, judicious medication might secure a more speedy recovery, as well as promote the comfort of the patient during the recuperative process. Hence, in those cases where anodynes appear to secure a rapid and complete recovery, we can not logically assume they would have done as well without the drug. Every medical man knows the value of *Opium* in the treatment of peritonitis, and few, I imagine, rely upon any other article, or combination of articles in which *Opium* is not included, administered internally, in the treatment of that disease in its early or active stage. Let me here throw in a disclaimer to the belief that peritonitis is a peculiar disease. It is a simple inflammation of the outer lining membrane of the bowels, differing in no respect whatever in its nature from simple inflammation of the pleura and other serous membranes. The phenomena of the ailment in the different localities, vary, because of the locality, or, rather, because of the difference of function in the structures in the different localities, and not because of difference in the functions of the membranes themselves, because they are one and the same wherever found, and the same treatment is applicable to all, except where other structures may contraindicate it. Now, if *Opium* is apparently of potential benefit in the treatment of one structure of the human system, why may it not be equally so of another? On the surface, there is nothing to exclude the presumption. Experience, practical observation, however, establishes the fact that *Opium* is not admissible in the treatment of certain organs, the brain, for instance, and the glandular system may put in a qualified claim to the same consideration; but experience raises no sufficient objection to its use in inflammation of the mucous membrane of the bowels.

Perhaps, inflammation of no structure or organ has had so much to do toward inducing my present train of thought, as that of the rectum; inflammation at that point being recognized by the erudite as colitis, colonitis, colo-rectitis, to say nothing of numerous other learned synonyms, and by the unlearned in medical lore, usually styled "the vulgar," as dysentery and bloody flux. Dysentery is characterized by mucous discharges mixed with blood, attended with burning pain at the anus, with severe, grinding, colicky pain higher up, and in many cases, with an almost constant and an urgent desire to evacuate the bowels; indeed, so urgent is the demand to go to stool as to be very nearly, if not quite, resistless. Now, when pain is encountered, so constant and distressing, the most obvious indication is to relieve it, and the profession, in this disease, are united upon that necessity. Various, perhaps, is the resort, but the majority exhibit *Opium*, or its preparations. This, in my opinion, is wise. But a too common impression prevails that other internal measures are necessary to remove the *cause* of the inflammation going on at the lower end of the gut, and a supposed sluggish liver is generally made the scape-goat to bear the sins originating elsewhere, and that much calumniated organ is persistently bedeviled to induce it to undo that which it had "neither art nor part" in doing. My experience has long since led me to the conclusion that dysentery, as a rule, is amenable to much simpler treatment than is involved in the idea that the liver is the faulty member and the portal circulation needing to be unloaded. Opiates in my hands have yielded the best results. In my young patients I promise myself less from this line of treatment; but even in them there is much to hope from it. Various circumstances, in adults as well as in infants, at times require a deviation from the rule, but, after satisfying myself that the upper bowels are not loaded, my practice is to obtund sensibility as rapidly as is safe, and hold the patient under the influence of the narcotic long enough to allow the inflamed membrane time to heal. 'Tis very true that the patient or his friends may become, and often do become, restive under the prolonged locking up of the bowels; but I ask them if they had an inflamed elbow, would they persist in bending it in order to heal it? This may be considered a palpable sort of argument to most minds, and usually I find no great difficulty in having my own way. A laxative may be resorted to finally, and yet, such is my confidence in the natural powers, that I not unfrequently omit it.

More than six years since, my wife was attacked with the most violent dysentery ever occurring in my family, and the only medicine I gave her was *Morph. Sulph.*, in half grain doses, every two hours, till a fair degree of comfort was obtained, when the dose was repeated at longer intervals, yet so as to keep up the impression for a week, the quantity being then decreased and the intervals lengthened; but, soon finding no increase of the disease, the medicine was suspended entirely. Within a fortnight from the commencement of the attack, the natural action of the bowels was resumed and the general health returned. I am safe in saying that was the most violently painful case of the disease I ever encountered. Comparative comfort was not secured till the second day, though some sleep was obtained, out of which she was not awakened to take medicine. Now, whether opiates, or the anodynes have a specific curative effect upon inflammation, wherever established, or not, I am convinced that in dysentery the judicious obtunding of sensibility in all cases where pain is a prominent feature, contributes to the recuperative efforts of nature. Except in very mild cases, fomentation over the abdomen of simple warm water, or of hops, or other bitter herbs, I rarely omit; though I confess to the belief that the addition of herbs other than hops to the water, is nearly "all in your eye." As they do no harm, however, whims of patients or their friends in this direction, may be safely gratified.

Injections I have resorted to infrequently, not hoping much from them: but, having learned of late the wonderfully curative effect of *Carbolic acid* in fresh wounds, whether incised or contused, I have thought it possible that article might be equally applicable in dysentery, sporadic, epidemic, or chronic, and am determined to try it topically in the first favorable case that comes under my care. The analogy between inflamed mucous membrane and fresh wounds is so remote as perhaps to cause the proposed treatment to appear ridiculous, but, nevertheless, I shall try it. The putrefactive tendency of epidemic dysentery is so conspicuous, that the antiseptic properties of *Carbolic acid* may here, when topically applied, find an appropriate field for usefulness.

